# Duet synchronization interventions affect social interactions

**DOI:** 10.1038/s41598-024-60485-w

**Published:** 2024-04-30

**Authors:** Valentin Bégel, Alexander P. Demos, Caroline Palmer

**Affiliations:** 1 Department of Psychology, McGill University, Montreal, Canada; 2 Department of Psychology, University of Illinois at Chicago, Chicago, USA; 3 Present Address: Institut des Sciences du Sport-Santé de Paris (I3SP), Paris Cité University, 1 Rue Lacretelle, 75015 Paris, France

**Keywords:** Cognitive neuroscience, Computational neuroscience, Human behaviour

## Abstract

Humans’ complex behavior, such as speech, music, or dance, requires us to coordinate our actions with external sounds as well as with social partners. The presence of a partner can influence individuals’ synchronization, and, in turn, social connection with the partner may depend on the degree of synchronization. We manipulated the synchronization quality in intervention conditions to address the causal relationship between observed temporal synchrony and perceived social interaction. Pairs of musician and nonmusician participants first performed a turn-taking task consisting of alternating which partner tapped their melody in synchrony with a metronome (each tap generated the next tone in the melody). In two intervention conditions, participants attempted to synchronize their melodies simultaneously with their partner, either with normal auditory feedback (normal feedback) or randomly placed delayed feedback on 25% of melodic tones (delayed feedback). After each intervention, the turn-taking condition was repeated, and participants completed a questionnaire about connectedness, relationship, and feeling of synchronization with their partner. Results showed that partners’ mean asynchronies were more negative following the delayed feedback intervention. In addition, nonmusician partners’ tapping variability was larger following the delayed feedback intervention when they had the delayed feedback intervention first. Ratings of connectedness, relationship, and feeling of synchronization with their partner were reduced for all participants after the delayed feedback Intervention. We modeled participants’ synchronization performance in the post-intervention turn-taking conditions using delay-coupling oscillator models. Reductions in synchronization performance after delayed feedback intervention were reflected in reduced coupling strength. These findings suggest that turn-taking synchronization performance and social connectedness are altered following short interventions that disrupt synchronization with a partner.

## Introduction

Humans can coordinate their actions with external sounds to produce complex behavior such as speech, music, or dance. These tasks are often performed with social partners, either simultaneously or taking turns with others^1–3^. Performing an auditory synchronization task with a partner (as opposed to a computer-generated sound) influences participants’ ability to synchronize because participants can mutually influence each other^4–6^. In addition, feelings of social connection with a partner depend on the amount of the partners’ synchronization and the degree of perceived synchrony^4,7^. Auditory-motor synchronization capacities are influenced by music practice and musical training^8–11^. Yet, little research has addressed the causality relationships between changes in auditory feedback from partners’ performances and their influences on subsequent synchronization and perceived social connection. Here, we manipulated the auditory feedback available during joint synchrony tasks as pairs of individuals performed simple melodies together, and we measured that impact on perceived social interaction and behavioral synchrony in subsequent turn-taking tasks. We addressed the causal relationship between the feedback interventions and their subsequent effects on synchrony and perceived social interaction; each pair performed each intervention condition followed by a joint synchrony condition.

Turn-taking is a natural feature of social interactions such as conversations, music-making, or dancing. It is observed in specific types of music performance common in genres such as Western jazz and improvisation, in which musicians alternate performing rhythmic and melodic phrases (one starts after the other has finished). Turn-taking tasks offer powerful designs for evaluating how individuals learn by testing the influence of hearing one’s partner perform the same task^1,2,4,5,12^. A partner’s presence can alter a musician’s performance, compared with learning the same task in a solo or synchronous situation. Turn-taking may enhance awareness of one’s partner and deliver social benefits; a study of turn-taking in adolescents resulted in greater empathy than ratings following synchronous (simultaneous) tasks^13^.

Another variable influencing music performances, such as turn-taking, is the similarity between partners’ optimal rhythmic performance rates in the absence of any auditory cues or instructions, called Spontaneous Production Rate (SPR). Partners with similar spontaneous rates synchronize better than partners with different rates, in both simultaneity tasks^6,14^ and in turn-taking tasks^4^. Partners tend to drift in tempo toward their spontaneous rates when performing at a different rate^4^.

Musical skills in synchronization tasks are accompanied by a decreased reliance on auditory feedback. Whereas nonmusicians tend to be highly influenced by altered feedback, individuals with greater musical training tend to perform equally accurately in the presence or absence of feedback^15^ and are less disrupted by altered auditory feedback^16,17^. Long-term musical practice enhances synchronization skills, as individuals with many years of experience and training display superior rhythmic performance compared to nonmusicans^9,10^. Improving participants’ rhythmic skills with short-term rhythmic interventions is also possible^8^. Intervention studies using music have primarily focused on training in neurological or neurodevelopmental diseases that affect rhythmic skills, such as Parkinson’s Disease^18^ or Dyslexia^19^. These interventions usually involve rhythmic tasks such as finger tapping or synchronizing gait with an auditory cue and are carried out over a few weeks, with several training sessions per week. Some intervention studies also involve a social component, such as dancing with a partner or in groups^4,20–22^. We test whether short-term interventions that rely on auditory feedback will change synchronization accuracy in individuals with and without musical training.

Auditory feedback interventions that modulate partners’ synchronization may in turn influence social affiliations with a partner. Social affiliation observed in musical ensembles may be partly due to action synchronicity, as individuals who engage in synchronous behavior tend to show increased social connections^23,24^ and positive feelings toward their partners^25^. Several studies have reported that the degree of perceived synchrony in a joint musical task, rather than the observed behavioral synchrony, was related to subsequent interpersonal liking between partners^7,26^. These results suggest that interventions that disrupt normal auditory feedback may also disrupt social affiliation with one’s partner. In this study, we measure the consequences of the intervention condition on synchronizers affiliative responses using self-report social questionnaires.

What mechanisms account for musical synchronization? Musical synchronization requires individuals to anticipate the timing of future auditory cues based on information perceived previously in the sequence. This often results in anticipatory behavior that can be modeled using nonlinear oscillators, such as in delay-coupled systems^27–29^ (for a review see^30^). For example, the synchronization of a person with a metronome can be represented as two oscillators, each with a natural (spontaneous) frequency of oscillation (intrinsic frequency). The driven oscillator (the human) receives instantaneous feedback from the driver oscillator (the metronome) and compares it with feedback from its own state at a time delay. The coupling of the driven oscillator to the driver is based on two parameters: a coupling strength and a time–delay parameter^31^. Greater coupling is necessary to achieve synchronization when the difference between the intrinsic frequency of the two oscillators is large. Supporting evidence is seen when partners with large differences in the spontaneous rates at which they produce a melody try to synchronize with each other, compared with partners with smaller differences^6,32,33^. The time delay parameter, thought to reflect neural transmission rates^34^, is usually kept at a constant value for ease of modeling the participants’ behavior.

Delay-coupled models have also been applied to two-person synchrony tasks^29^ in which two coupling terms are introduced to capture the coupling of Partner A to Partner B and vice versa, referred to as bidirectional coupling. Delay-coupled models have also been applied to two individuals participating in a turn-taking paradigm, in which the coupling terms represent Partner A’s coupling with the metronome and Partner B’s coupling with a metronome^4^. The intrinsic frequency of the driven oscillator represents the participant’s SPR estimated by the delay-coupling model^4^. Interestingly, partners who showed greater coupling terms in the delay-coupled model, and thus tighter synchrony with their partner, gave higher ratings of pleasantness and relationship with their partner. This finding suggests that coupling strength contributes to the degree of interpersonal liking. However, it is still unknown whether coupling strength can be affected by short-term feedback interventions and if the changes in coupling strength reflect the changes observed in interpersonal liking.

In this study, we tested the effect of short-term auditory feedback interventions on musicians and nonmusician partners’ subsequent synchronization abilities and coupling strength in a turn-taking task (alternating which partner synchronizes with the metronome) and social interaction ratings. Participants (musically trained and untrained) produced simple melodies simultaneously (interventions) or while taking turns with a partner. In this novel task, partners tapped on a force sensor and each tap produced the next tone in a familiar melody, allowing comparisons between musicians and nonmusicians. Participants first tapped their melody at a comfortable consistent rate (Spontaneous Production Rate, SPR), a measure used to estimate their intrinsic frequencies. Two auditory feedback interventions consisted of normal auditory feedback, or randomly placed delayed feedback that sounded 30–70 ms later than the participants’ taps. After each intervention condition, participants completed social interaction questionnaires and then performed the turn-taking task in which they synchronized with a regular auditory metronome. We hypothesized that the social interaction ratings and synchronization performance in the turn-taking task would be poorer after the delayed feedback intervention condition than after the normal feedback intervention. We also expected that the effect of the interventions on synchronization and social interactions would be reduced in musically trained participants, as they rely less on feedback than nonmusicians. The computational model was fit to partners’ asynchronies in the turn-taking task to estimate the two parameters of partners’ coupling strength and natural frequency differences while holding the time delay constant across participants. We predicted that coupling strength across the turn-taking conditions should be larger for musicians than for nonmusicians due to the musicians’ experience adapting to their partner.

## Methods

### Participants

Forty-eight participants were recruited. Half of the participants (Musicians, 21 women; age range: 18–31 years, *M* = 21.87, *SD* = 2.80), had at least six years of private instrumental musical training (range: 6–16 years, *M* = 10.06, *SD* = 2.96). The other half (Nonmusicians, 16 women; age range: 19–34 years, *M* = 24.21, *SD* = 4.53), had less than two years of private instrumental musical training (range: 0–1.5 years, *M* = 0.37, *SD* = 0.52). Participants who reported a neurological condition were not included in the study. All participants exhibited normal hearing in the frequency range of stimuli used in the experiment (< 30 dB HL for single tones in the 125–750 Hz pitch range), tested with a Maico MA40 audiometer. Musicians were randomly paired with Musicians, and Nonmusicians were randomly paired with Nonmusicians. Participants received a small fee for their participation. The McGill University Research Ethics Board approved the study. The study was conducted in accordance with the Declaration of Helsinki.

### Equipment and stimuli

A force-sensitive resistor (FSR) controlled with Arduino and connected to a Linux computer (Dell T3600 running Fedora 16) by a MIDI cable via USB was used to record participants’ finger tapping. Each tap produced a tone in a melody heard through headphones (AKG K240 Studio) that was sounded on a Roland Studio Canvas SD-50 tone generator with a marimba timbre (GM2, patch #13, bank #0). The FTAP program (version 8^35^) controlled the presentation timing of the sounds and recording of the taps, ensuring a negligible measurement delay from the start of the tap on the pad to the production of the sound (< 2 ms, see Supplemental material in^33^).

Spontaneous Rate task, Solo synchronization task and the Joint turn-taking tasks were based on a novel 8-note ascending and descending melody corresponding to a G major pattern (G3, A3, B3, C4, D4, C4, B3, A3) for partner A’s feedback and the same pattern one octave higher for partner B’s feedback (G4, A4, B4, C5, D5, C5, B4, A4). Each partner received the same melody in a different octave to avoid confusion as to who was producing which pitches. This simple scale-based melody was chosen for its familiarity among participants regardless of their musical training. Metronome beats were presented with a high-pitched woodblock timbre (Roland Studio Canvas GM2, patch #116, bank #0).

The auditory feedback presented over headphones corresponded to the melody in 3 tasks: Spontaneous Production Rate, Solo synchronization (tapping with the metronome), Joint turn-taking synchronization (alternating with a partner tapping with the metronome). In a fourth condition (Intervention task), partners tapped simultaneously and heard the feedback from both partners’ melodies. The auditory feedback was either normal or delayed in time by 30-70 ms randomized around the mean of 50 ms. Twenty-five percent of the serial positions were delayed, half of which were in one partner’s taps, and the other half were in the other partner’s melodie tones. Delayed feedback was only introduced to one part at a time. Therefore, each partner experienced the same mean delay placed at different positions.

### Design

**Figure 1:**
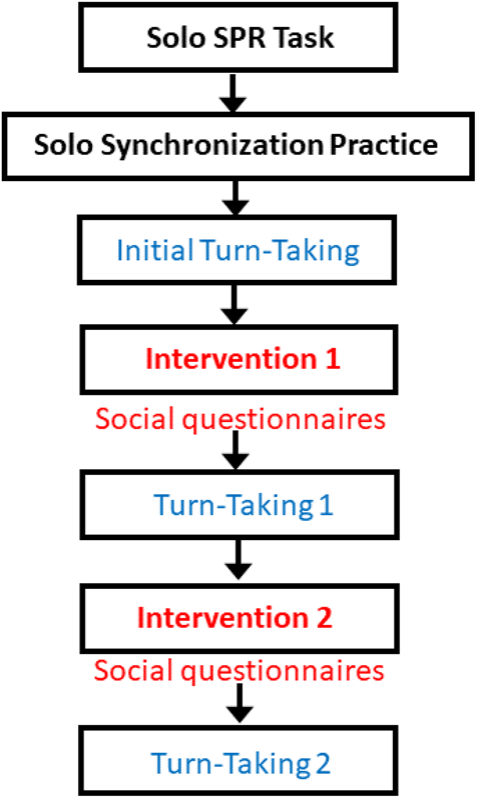
Depiction of order of conditions. Intervention conditions refer to synchronization performance with a partner during Normal or Delayed auditory feedback. Intervention 1 was the Normal feedback condition and Intervention 2 was the Delayed feedback condition for half of the pairs; the other half had the Intervention conditions in the opposite order. Turn-taking conditions refer to partners taking turns synchronizing with an auditory cue.

The experimental tasks, ordered as in Fig. 1, consisted of a Spontaneous Production Rate (SPR) Solo task, Joint turn-taking tasks (turn-taking with a partner), and Intervention tasks (synchronized tapping with a partner). In one Intervention condition (Delayed Feedback), the auditory feedback was delayed as described above. In the other Intervention condition (Normal Feedback), normal auditory feedback was provided for all taps. After each Intervention, the partners performed the Joint turn-taking again. The comparison of the Joint turn-taking tasks (Post-normal Intervention and Post-Delayed Intervention) allowed a test of the impact of the interventions on subsequent solo synchrony with a metronome (without a partner). The order of the two Intervention conditions was counterbalanced across pairs: half of the pairs performed the Normal Feedback Intervention first, and the other half performed the Delayed Feedback Intervention first. Thus, the Intervention factor (normal/delayed auditory feedback) was manipulated as a within-subject variable; the Group factor of musical expertise (Musician/Nonmusician) was a between-subject variable.

### Procedure

Two participants were randomly assigned to the same time slot, forming pairs. None of the pair members (partners) knew each other prior to the experiment. Upon arrival at the lab, participants gave informed consent and completed an audiometry screening followed by the SPR task and the Solo synchronization task in different rooms.

Participants were first familiarized with the Arduino pad on which they tapped to produce the tones of the 8-note melody which they heard over headphones. They heard it twice during this practice trial. Then they completed the SPR tapping task, in which they were instructed to tap the melody at a steady and comfortable rate, without stopping between repetitions until the taps generated no more auditory feedback. Each trial consisted of tapping the melody 4 1/2 times in the absence of any rhythmic pacing cue. Each participant performed one practice trial and three experimental trials of the SPR task. Next, participants were asked to complete a short musical background questionnaire while the experimenter computed the participant’s mean SPR. The rate of the auditory cue in all subsequent conditions was set to the mean of the two partners’ mean inter-tap intervals (ITIs) from the SPR task.

Next, each participant completed the Solo synchronization task during which an auditory pacing cue was sounded at the mean SPR of the two partners. Each participant first performed a practice trial in which they were instructed to tap the 8-note melody twice without stopping between repetitions while synchronizing with the auditory cue. Then, each participant practiced tapping the melody for 8 beats in synchrony with the auditory pacing cue and then waited 8 beats. They alternated tapping for 8 cued beats and waiting for 8 cued beats until the trial ended (mimicking their part in the turn-taking task). Each participant completed three practice trials in which they heard the auditory cue and their own tapping feedback on all trials.

**Figure 2:**
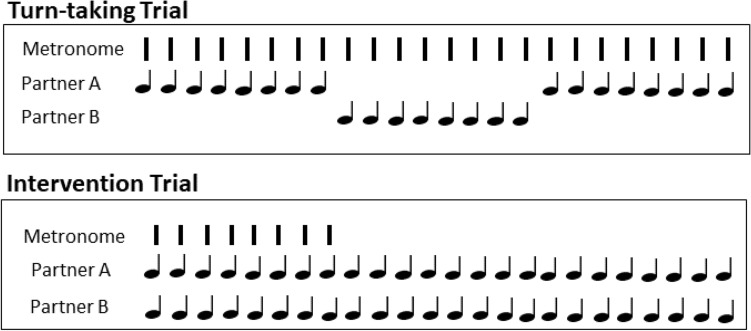
Sample turn-taking and intervention trial. Partners synchronized with each other during Intervention trials and with the auditory cue during Turn-taking trials.

The two partners then performed the Joint turn-taking task in the same room where they sat facing each other at separate tables containing a force-sensitive Arduino pad. A screen was placed between the partners so that they could only see their partner’s head and shoulders, to avoid influences of the partners’ finger movements. The partners performed a practice trial, alternating tapping their melody without a pacing stimulus. Then, they performed a practice turn-taking trial in which one partner was instructed to start tapping after eight metronome beats and the other partner started synchronizing when the first partner stopped, alternating their taps until the sounded feedback ended. An example of the trial structure is shown in Fig. 2. Each trial lasted 8 taps × 2 partners × 4 repetitions or 64 beats in total (joint turn-taking trials contained the same number of taps per partner and the same total length as in the solo synchronization trials). Partners completed one practice trial and three experimental trials of the joint turn-taking task.

Next, participants were presented with one of the Intervention conditions (Normal or Delayed Auditory Feedback). In each Intervention condition, the two partners were instructed to start tapping simultaneously after eight metronome beats (see Fig. 2). After eight more beats, the auditory cue stopped and the partners were instructed to continue synchronizing their taps with their partner while maintaining the cued rate. After the first Intervention condition (Normal Feedback or Delayed Feedback), participants repeated the Joint turn-taking condition again. Then, they completed the second Intervention condition, followed by the Joint turn-taking condition again. Before each intervention and Joint condition, the partners performed one practice trial to re-orient themselves to each task. After each Intervention condition (see Fig. 2), participants completed a questionnaire with three questions presented on a 7-point Likert scale: how connected they were with their partner (connectedness; 1 = not connected to 7 = fully connected^36^), how close their relationship was with their partner using overlapping circles (relationship^37^), and how successful they thought their synchronization was with their partner (synchronization; 1 = not synchronized, 7 = fully synchronized^36^). The experiment lasted approximately 75 min.

### Data analysis

**Figure 3:**
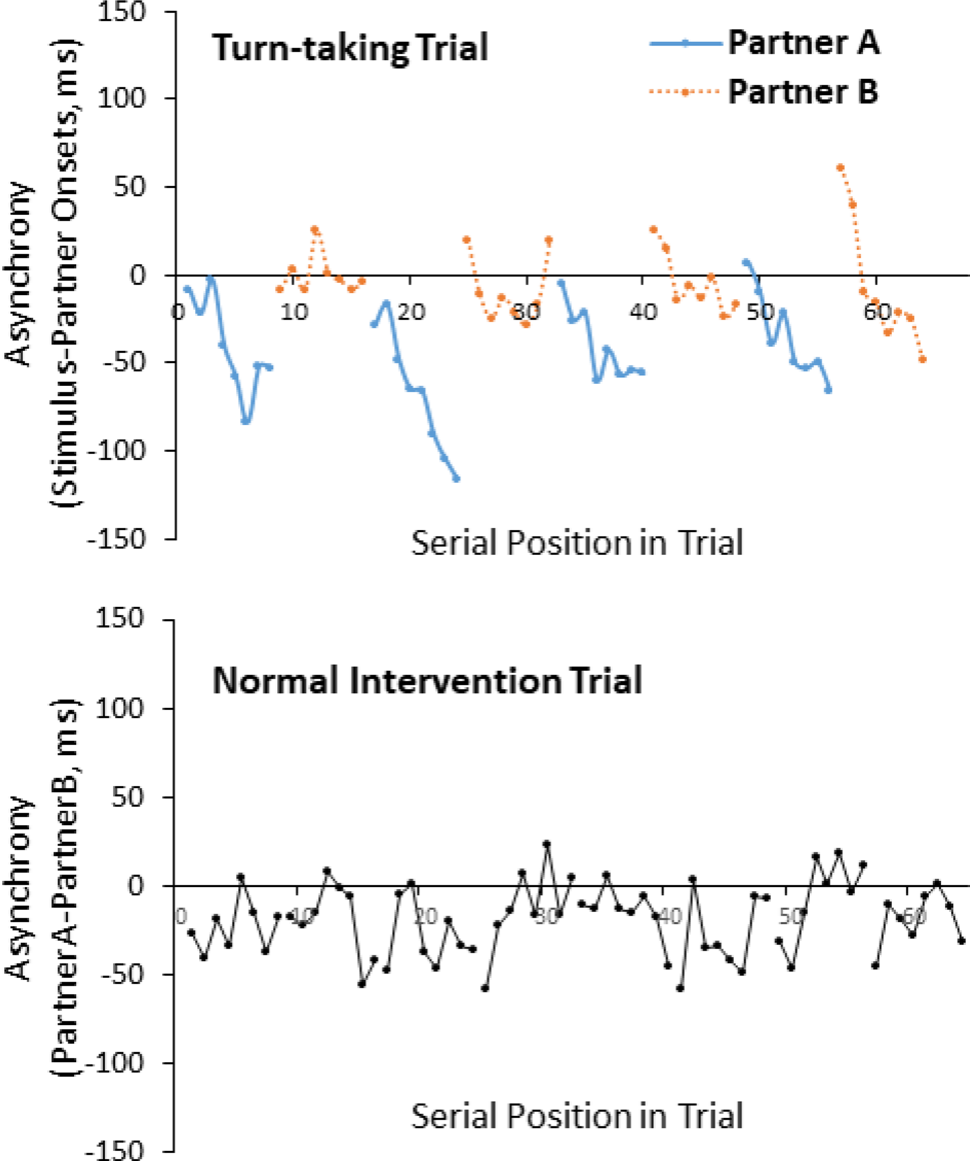
Sample turn-taking and intervention trial (asynchronies).

As in previous studies, each participant’s mean rate in the SPR task was computed as the mean ITI (in ms) across the middle two repetitions (most stable sections) of each experimental trial^4,6^. The Coefficient of Variation (CV) in the SPR task was also calculated on the same taps ([SD ITI/mean ITI]). Each tap in the Solo and Joint turn-taking trials (synchronization with an auditory cue) was matched with the nearest cued beat following a nearest-neighbour approach^10^. The graphs of the asynchronies for each trial were then visually examined to ensure that the nearest-neighbour algorithm yielded correct results. Signed asynchrony values were computed as the participant’s tap onset time minus the auditory cued onset time in ms. Hence, a negative asynchrony value indicated that the participant tapped before the cue, while a positive value indicated that the tapper lagged behind the cue. A sample trial of a turn-taking and normal intervention task is shown in Fig. 3. The SD of the signed asynchronies was also calculated.

### Delay-coupled model

A unidirectional delay-coupled model similar to the one used in^4^ was fit to the mean asynchronies (participants’ taps minus the auditory cued onset) in the turn-taking conditions. A driven oscillator (the participant) coupled with (was influenced by) a driver oscillator (the auditory cue) that is not influenced by the driven oscillator. The driver and driven oscillator’s behavior can be described in terms of relative phase by the equations below^29,31^.

1 $$\begin{gathered} \dot{\theta }_{1} = \omega_{1} \hfill \\ \dot{\theta }_{2} = \omega_{2} + \kappa \left( {\theta_{1} - \theta_{2,\tau } } \right) \hfill \\ \end{gathered}$$

In this equation, $\dot{\theta }_{1}$ represents the phase of the driver metronome and $\dot{\theta }_{2}$ represents the phase of the driven oscillator (the participant’s tapping). $\omega_{1}$ and $\omega_{2}$ denote the intrinsic frequencies of the two oscillators. $\omega_{1}$ corresponds to the auditory metronome rate. $\omega_{2}$ was modeled as $\omega_{diff}$, the signed difference between the driven oscillator’s intrinsic frequency and the cued metronome rate ($\omega_{diff}$ = $\omega_{2}$- $\omega_{1}$). The driven oscillator couples with the driver oscillator via the coupling term κ, that influences the difference in relative phase between the oscillators.

The model-fitting procedure consisted first of a global parameter search using a genetic algorithm, after which the obtained parameters were passed to a local search algorithm (constrained nonlinear multivariate function). In all model simulations, the initial conditions for the first fitting to constraint the global space were randomly chosen from within the specified range for each parameter ($\kappa$ = 0–50; $\omega_{diff}$ = − 300 to 300; $\tau$ = 0–50). The optimized values from this Genetic Algorithm formed the initial parameter values for the second stage^4,29^. The error function was a weighted sum-of-squared-errors function. The first serial position was weighted by four and the eighth (last) position was weighted by two, similar to^4,29^. We fit the model 10 times on the averaged series of 8 asynchronies for each condition and for each participant. The model whose parameters generated the best (lowest) root mean squared error (RMSE) was chosen for subsequent analysis.

The driven oscillator’s three parameters were allowed to vary in the first fit of the model with following ranges: $\omega_{2}$ was allowed to vary within a range of ± 300 relative to the value of the $\omega_{1}$ parameter (auditory cued rate set to each pair’s mean SPR) to incorporate the largest observed difference between partners’ SPRs, $\kappa$ was allowed to vary from 0 (no coupling) to 50 ms, and $\tau$ was allowed to vary with a range of 0 (no time delay) to 50 ms. $\omega_{2}$ was modeled as the signed difference between the partner’s intrinsic frequency and the cued metronome rate, $\omega_{diff}$ ($\omega_{diff} = \omega_{2} - \omega_{1}$). Following these initial model fits, we fit the model again, using a fixed time delay $\tau$ set to the median $\tau$ value (21 ms) across participants from the initial model fits. As in^4^, the value of the time delay parameter $\tau$ was held constant as it is assumed to reflect neural transmission delays^34,38^. Thus, the final model was fit on two parameters, $\kappa$ and $\omega_{diff}$. $\kappa$ and $\omega_{diff}$ parameters were again allowed to vary within 0–50 ms and ± 300 ms, respectively.

### Statistical analyses

All analyses were run in R Statistical Software (v4.3-1^39^). Tests of the behavioral measures included analyses of variance (ANOVA; afex v1.0-1^40^). Linear contrasts were run with the emmeans package (1.7-2^41^) and *p*-values were corrected within family when there were more than two tests using a Tukey correction.

Linear Mixed models were applied using the lme4 package in R (v1.1-8^42^) and Type III F-tests are reported with Saitherwaith degrees of freedom using the LmerTest package (v3.1-3^43^).

## Results

### Spontaneous production rates

**Figure 4:**
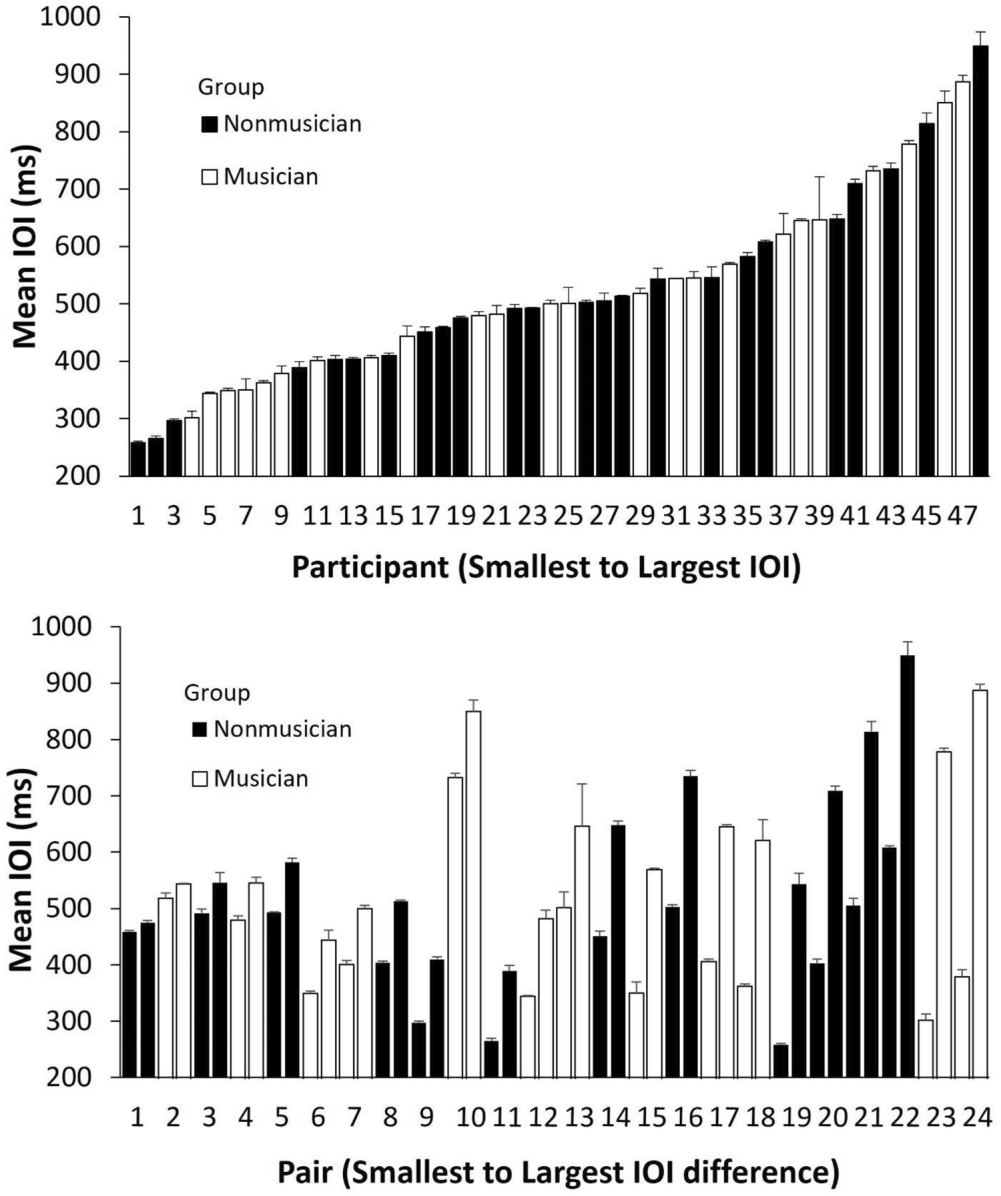
Mean individual Spontaneous Production Rates. Error bars indicate standard errors of the mean. Top: Individual participants ordered from fastest to slowest. Bottom: (Randomly assigned) pairs ordered from smallest difference between partners’ SPRs to the largest difference.

The SPR value (representing an intrinsic frequency) was computed for each participant as the mean ITI across three trials of the Solo production task. The distribution of SPR across participants, ordered from fastest to slowest, is presented in Fig. 4 (top). Figure 4 (bottom) shows the distribution of pairwise differences in partners’ SPRs, ordered from the smallest pairwise difference to the largest difference. We assessed whether mean SPRs and tapping variability (CV of ITIs) differed between musicians and nonmusicians with one-way ANOVAs by Group (Musician, Nonmusician). No main effect of musical training was obtained on mean SPR, *F*(1, 46) = 0.02, *p* = 0.88, $\eta_{G}^{2}$ < 0.001 (Musician, *M* = 526 ms, *SD* = 163; Nonmusician, *M* = 518 ms, *SD* = 166) or on CV of ITIs, *F*(1, 46) = 1.38, *p* = 0.24, $\eta_{G}^{2}$ = 0.029 (Musician, *M* = 0.050, *SD* = 0.042, Nonmusician, *M* = 0.67, *SD* = 0.055), indicating that musicians and nonmusicians did not differ in mean tempo or motor variability when they tapped in the absence of a metronome cue. Similar to previous studies, large individual differences were observed (fourfold difference from the fastest individual to the slowest individual), providing a good test of the intrinsic frequency parameter in the model fits.

### Group differences in initial turn-taking

We examined group differences in synchronization accuracy and variability in the initial turn-taking task. A one-way ANOVA by group (Musician, Nonmusician) on mean asynchronies revealed a main effect of music training, *F*(1, 46) = 4.19, *p* = 0.046, $\eta_{G}^{2}$ = 0.083. Musicians tapped closer to the beat on average (*M* = − 21.58, *SD* = 16.29) than nonmusicians (*M* = − 38.45, *SD* = 36.91). Musicians’ asynchronies were also less variable, indicated by their smaller SD of asynchronies (*M* = 23.30, *SD* = 9.55), than nonmusician (*M* = 54.10, *SD* = 31.01), confirming that the musically trained individuals differed from the untrained individuals, *F*(1, 46) = 21.52, *p* < 0.001, $\eta_{G}^{2}$ = 0.319.

### Intervention effects on synchrony

Next, the effects of the auditory feedback interventions on partners’ simultaneous synchronization were assessed. A three-way ANOVA on mean asynchronies by intervention condition (Normal, Delay), group (Musician, Nonmusician), and intervention order (Normal first, Delay first) showed no significant main effects or interactions. The same analysis applied to the SDs of asynchronies showed a main of intervention condition, *F*(1, 20) = 28.42, *p* < 0.001, $\eta_{G}^{2}$ = 0.409. Both groups synchronized more variably in the delayed feedback condition (*M* = 59.50, *SD* = 24.04) than in the Normal feedback condition (*M* = 30.29, *SD* = 7.03). No other main effects or interactions were obtained.

### Intervention effects on social interaction

We tested the effects of the interventions on the social interaction ratings that followed each intervention. A three-way ANOVA on the connectedness ratings by intervention condition (Delay, Normal), group (Musician, Nonmusician), and intervention order (Delay first, Normal first) showed a main effect of intervention condition, *F*(1, 44) = 6.39, *p* = 0.015, $\eta_{G}^{2}$ = 0.025. Participants rated their partner as more connected to them after the normal feedback intervention (*M* = 4.69, *SD* = 1.15) than after the delayed feedback Intervention (*M* = 4.31, *SD* = 1.27). There were no other significant main effects or interactions. The perceived Synchronization ratings also indicated a main effect of Intervention condition, *F*(1, 44) = 3.46, *p* < 0.001, $\eta_{G}^{2}$ = 0.110. Participants’ synchronization ratings were higher after the normal feedback intervention (*M* = 4.90, *SD* = 1.22) than after the delayed feedback condition (*M* = 4.09, *SD* = 1.19). There were no other main effects or interactions on the synchronization ratings.

**Figure 5:**
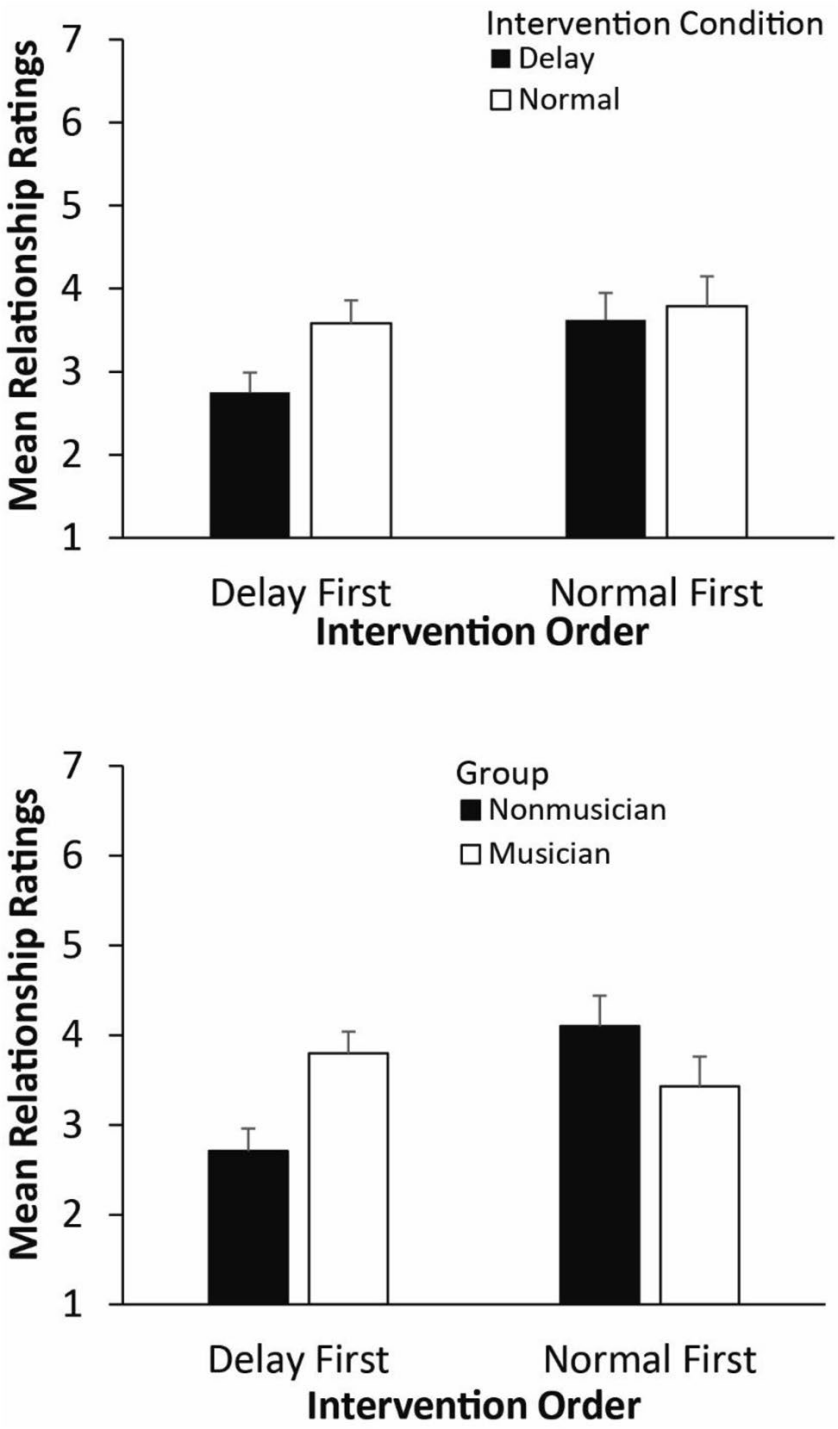
Mean social relationship ratings after Interventions. Top: by Intervention condition and Intervention order; Bottom: by Group and Intervention order. Error bars indicate standard errors of the mean.

The same analysis on the relationship ratings also indicated a main effect of Intervention condition, *F*(1, 44) = 11.99, *p* = 0.001, $\eta_{G}^{2}$ = 0.031, and a significant interaction between Intervention condition and Intervention order, *F*(1, 44) = 4.32, *p* = 0.044, $\eta_{G}^{2}$ = 0.011. As shown in Fig. 5 (top), relationship ratings were lower after the delay intervention when participants had the delayed feedback intervention first, compared with participants who had the normal feedback intervention first, *t*(44) = 3.92, *p* < 0.001, *d* = 1.15. The group x intervention order interaction was also significant, *F*(1, 44) = 4.87, *p* < 0.05, $\eta_{G}^{2}$ = 0.089, as shown in Fig. 5 (bottom). Relationship ratings were lower for the nonmusician group when they had the delayed intervention first, *t*(44) = 2.46, *p* = 0.019, *d* = 0.72; no significant difference by intervention order was found for the musicians.

Overall, the three social interaction questions (Connectedness, Synchronization, Relationship) indicated that participants were sensitive to the Intervention condition. Intervention order (Delayed feedback first) yielded lowest relationship ratings, especially for nonmusician participants. We repeated the analyses above with gender as a covariate; participant gender did not affect the results reported here.

### Intervention effects on turn-taking

We assessed the effects of the intervention conditions on synchronization accuracy and variability in the turn-taking task. A three-way ANOVA by intervention condition (post-Delay, post-Normal), group, and Intervention order (Delay first, Normal first) on mean asynchronies revealed a main effect of intervention condition, *F*(1, 44) = 7.40, *p* = 0.009, $\eta_{G}^{2}$ = 0.020. Mean asynchronies were larger (more anticipatory) in the turn-taking task that followed the delayed feedback intervention (*M* = − 38.50, *SE* = 5.43) than the normal feedback intervention (*M* = − 29.99, *SE* = 4.24). No other main effects or interactions reached significance.

**Figure 6:**
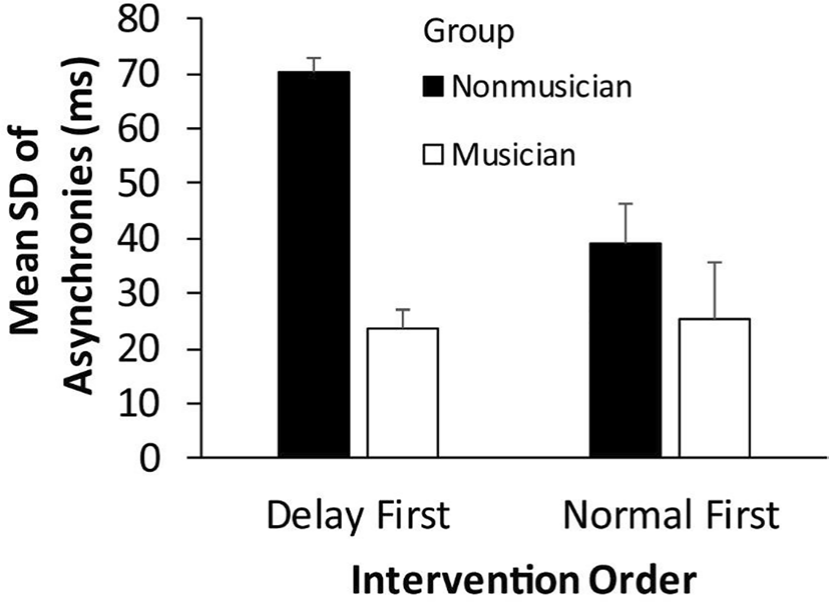
Mean SD of signed asynchronies in the Turn-taking task by Group and Intervention order. Error bars indicate standard errors of the mean.

Intervention condition effects were also seen in the SD of asynchronies in the post-intervention turn-taking tasks. The same ANOVA by Intervention condition (post-Delay, post-Normal), group, and intervention order (Delay first, Normal first) showed a significant main effect of froup, *F*(1, 44) = 20.29, *p* < 0.001, $\eta_{G}^{2}$ = 0.268, indicating that musicians (*M* = 24.54, *SD* = 10.57) were less variable than nonmusicians (*M* = 57.25, *SD* = 37.75). There was also a significant main effect of intervention order, *F*(1, 44) = 4.91, *p* = 0.032, $\eta_{G}^{2}$ = 0.081 (Delay first, *M* = 50.85, *SD* = 39.63; normal first, *M* = 30.94, *SD* = 17.61), and a significant interaction between group and intervention order, *F*(1, 44) = 6.11, *p* = 0.017, $\eta_{G}^{2}$ = 0.099. As shown in Fig. 6, nonmusician participants who had the delayed feedback intervention first showed larger SDs during turn-taking than any other group (Musicians with delayed feedback first, *t*(44) = 4.93, *p* < 0.0001, *d* = 1.44; Nonmusicians with normal feedback first, *t*(44) = 5.20, *p* < 0.0001, *d* = 1.39; Musicians with normal feedback first, *t*(44) = 3.31, *p* = 0.001, *d* = 0.97). Thus, the main effect of intervention order was mostly driven by the Nonmusicians. Overall, presenting the delayed feedback condition first affected nonmusicians’ tapping variability in the subsequent turn-taking more than the normal feedback condition. There were no other significant main effects or interactions.

### Delay-coupled model fits: turn-taking

**Figure 7:**
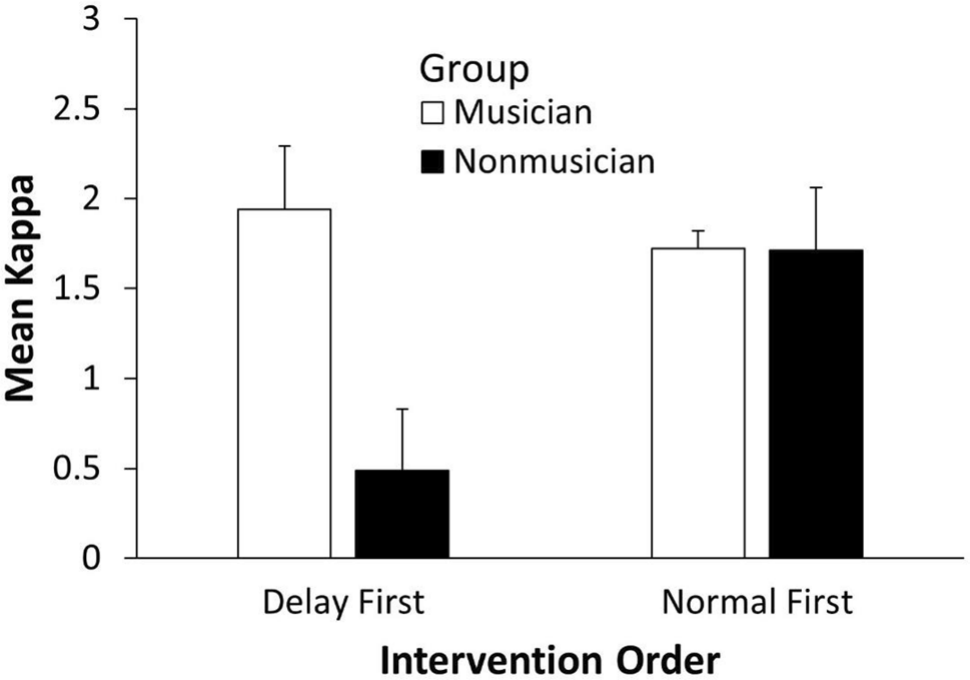
Mean kappa (coupling) values applied to the asynchronies from the Turn-taking task by Group and Intervention order. Error bars indicate standard errors of the mean.

The partners’ asynchronies from the post-intervention turn-taking tasks were fitted with the model shown in Eq. (1). We first tested the estimated model parameter kappa ($\kappa$) in a three-way ANOVA by intervention condition (post-Delay, post-Normal), group (Musician, Nonmusician), and intervention order (Delay first, Normal first). A main effect of group was observed, *F*(1, 44) = 4.23, *p* = 0.046, $\eta_{G}^{2}$ = 0.063. As predicted, $\kappa$ was larger for Musicians (*M* = 1.81, *SE* = 0.24) than for nonmusicians (*M* = 1.00, *SE* = 0.18). The ANOVA also revealed a significant interaction between Group and Intervention order, *F*(1, 44) = 4.09, *p* = 0.049, $\eta_{G}^{2}$ = 0.060, presented in Fig. 7. The Nonmusician group had larger $\kappa$ values for asynchronies in the turn-taking when the normal intervention condition occurred first than when the delay intervention was first, *t*(44) = 2.42, *p* = 0.019, *d* = 0.64. Among participants who had the delay condition first, musicians had larger $\kappa$ values than nonmusicians, *t*(44) = 2.88, *p* = 0.006, *d* = 0.84. The same analyses of the model parameter estimates of $\omega_{diff}$ (the intrinsic frequency values) showed no significant effects.

As other parameters can influence the $\kappa$ parameter values in $\tau$ the delay-coupled model, we compared the $\kappa$ and $\omega_{diff}$ values for each individual. Individual $\kappa$ and $\omega_{diff}$ values were correlated in each of the turn-taking conditions following the normal Intervention (Musicians, *r* = 0.88, *t*(22) = 9.06, *p* < 0.0001 and Nonmusicians, *r* = 0.52, *t*(22) = 2.84, *p* = 0.01), and the delay intervention (Musicians, *r* = 0.70, *t*(22) = 4.62, *p* < 0.001, and nonmusicians, *r* = 0.66, *t*(22) = 4.19, *p* < 0.001). Therefore, we tested whether the kappa differences found in the Turn-taking conditions held independently from the $\omega_{diff}$ values by fitting the same model while keeping the value of the $\omega_{diff}$ parameter fixed across participants, using the median value of the previous fit (25). Thus, only the coupling strength parameter $\kappa$ was allowed to vary. The same three-way ANOVA by Intervention (post-Delay, post-Normal), Group (Musician, Nonmusician), and Intervention order (Delay first, Normal first) was applied to the resulting $\kappa$ values. The interaction between Group and Intervention order remained significant after fixing $\omega_{diff}$, *F*(1, 44) = 4.48, *p* = 0.040, $\eta_{G}^{2}$ = 0.086.

Second, the model fit to individual differences in $\kappa$ were repeated with $\omega_{diff}$ included as a covariate in a mixed model. After controlling for $\omega_{diff}$, the main effect of group, *F*(1, 48.67) = 3.51, *p* = 0.067, the main effects of intervention order, *F*(1, 43.34) = 4.50, *p* = 0.039 (delay first, *M* = 0.74, *SD* = 0.46; normal first, *M* = 1.08, *SD* = 0.75), and the interaction between Group and Intervention order, *F*(1, 48.68) = 3.27, *p* = 0.077, were at or close to significance. Values of random effects were 0.39 (variance) and 0.62 (*SD*) for participants, and 0.07 (variance) and 0.62 (*SD*) participant|condition. The overall fit of the model was estimated using the conditional coefficient of determination as a measure of the effect size (conditional *R*^2^ = 0.79, marginal *R*^2^ = 0.62)^44^. Thus, the musicians’ larger coupling and the nonmusicians’ smaller coupling following the delayed feedback Intervention were not accounted for solely by $\omega_{diff}$ values.

### Correlations between the model’s parameter values and the turn-taking asynchronies

**Figure 8:**
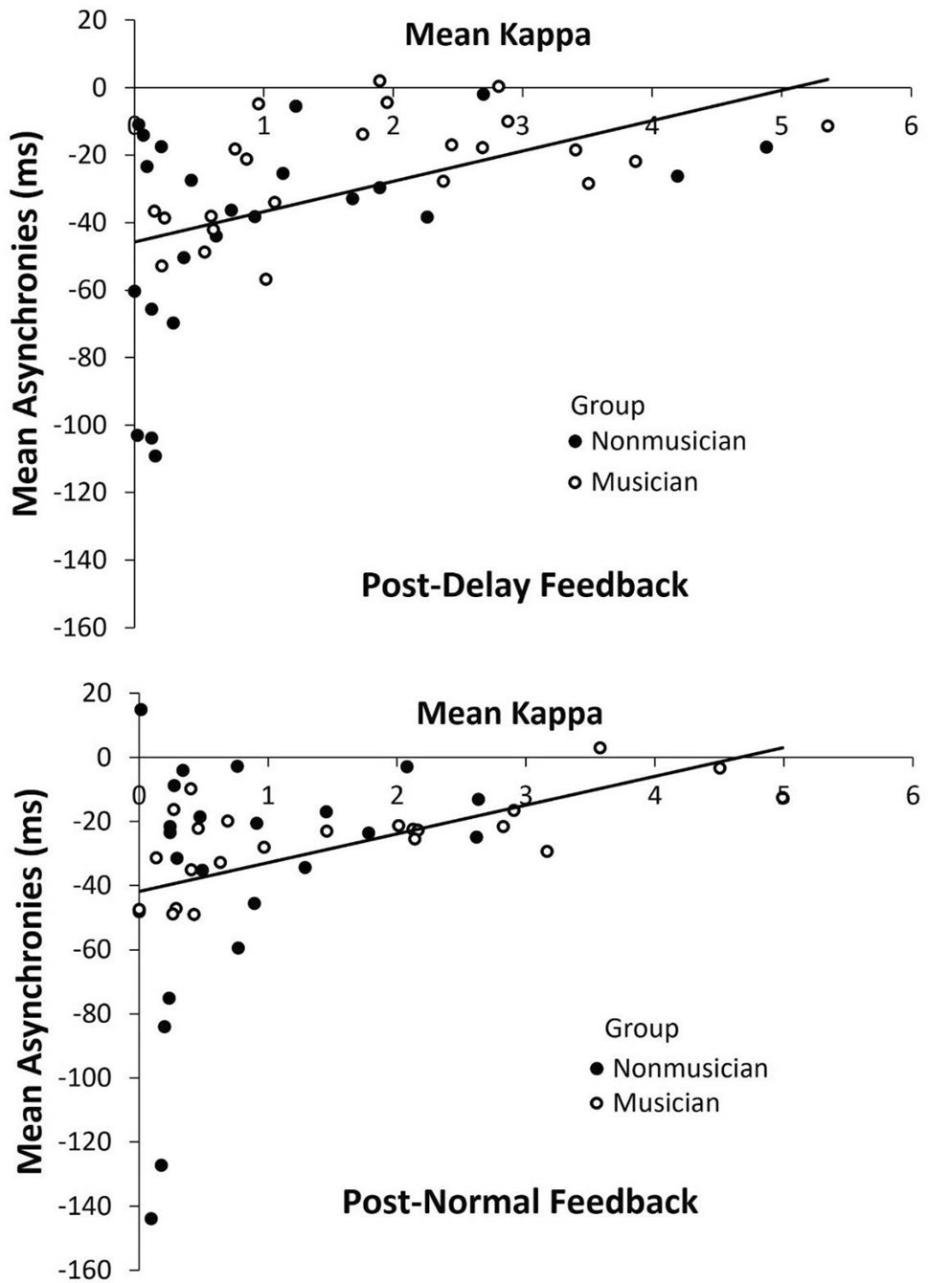
Correlations between kappa values from model fits and mean asynchronies in the Turn-Taking task. Top: Turn-taking condition following the Normal feedback intervention. Bottom: Turn-taking condition following the Delayed feedback condition.

We confirmed that the model $\kappa$ parameter reflected the synchronization accuracy in the Turn-taking task by correlating the values on an individual participant basis. Two outlier $\kappa$ values (defined as deviations greater than 3 SD from the mean) were excluded from these analyses. First, the correlations between $\kappa$ and mean asynchronies were computed, as shown in Fig. 8. As expected, positive correlations were observed between $\kappa$ and mean asynchronies in the Turn-taking conditions following the normal feedback intervention, *r* = 0.38, *t*(44) = 2.72, *p* = 0.009, and following the delayed feedback condition, *r* = 0.45, *t*(44) = 3.34, *p* = 0.002.

**Figure 9:**
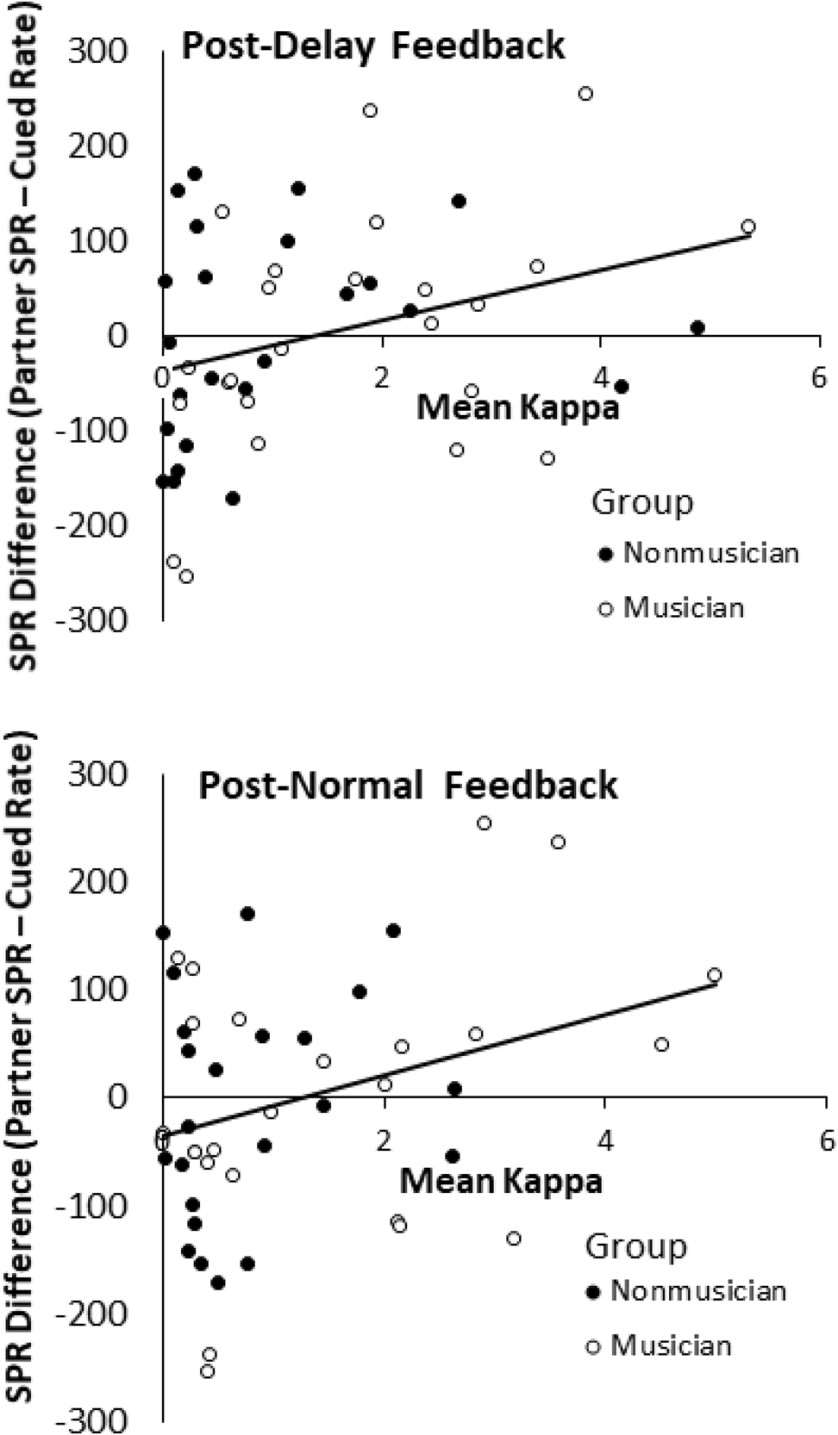
Correlations between individual kappa values from model fits to asynchronies in the Turn-Taking task and the difference in individuals’ SPR values minus the cued rate (in ms). Top: Turn-taking condition following the Normal feedback intervention. Bottom: Turn-taking condition following the Delayed feedback condition.

Second, we tested whether the coupling values increased for participants whose intrinsic frequencies (measured by the SPR) were farther from the auditory cued rate (the mean of the two partners’ SPR values). Correlations were computed between $\kappa$ and the difference defined by each participant’s SPR minus the cued rate (in ms). Thus, a positive correlation would indicate larger $\kappa$ when the participants’ intrinsic frequency was slower than the cued rate. As shown in Fig. 9, the correlations were significant in the turn-taking condition following the normal feedback intervention, *r* = 0.30, *t*(44) = 2.08, *p* = 0.043, and following the delayed feedback condition, *r* = 0.31, *t*(44) = 2.25, *p* = 0.029. Thus, the slower the participants’ SPRs were relative to the cued rate, the larger the coupling needed for the model to fit the turn-taking asynchronies.

## Discussion

Brief synchronization interventions affected partners’ perceived social interaction and subsequent synchronization in a turn-taking task. Both musically trained and untrained participants displayed more variable synchronization during the delayed auditory feedback intervention than the normal feedback intervention. Furthermore, subsequent perceptions of social connectedness, relationship, and synchronization with their partner were lower after the delayed feedback intervention than the normal feedback intervention. In addition, the partners’ synchronization in the subsequent Turn-taking task was disrupted when that task followed the delayed feedback intervention. Finally, the coupling and intrinsic frequency parameters in a delay-coupled model accounted for the changes in turn-taking synchronization following the different interventions. Thus, the brief interventions had causal outcomes on turn-taking synchronization, which could be reversed in a subsequent intervention for musicians and only partly reversed for nonmusicians who were affected by the order of the interventions.

Altered auditory feedback influenced partners’ synchronization as revealed by their increased tapping variability (i.e., less temporal stability) in the delayed feedback condition. Participants rated their synchronization as less successful in the delayed feedback than in the normal feedback condition, suggesting that they noticed the difference between the two conditions. This finding is consistent with previous reports of disrupted timing performance following delayed auditory feedback^45^. In our experiment, both musicians and nonmusicians’ tapping performance was affected, which contrasts with findings that musicians are less influenced by altered feedback^16,17^. However, in previous experiments, feedback was shifted by a constant value. In the current experiment, feedback shifts of random sizes and sequence locations occurred, making it impossible even for musicians to predict when and how the auditory feedback would be delayed and to adapt the timing of their taps accordingly. In addition, we used two-person synchronization tasks in which both participants’ taps were delayed, whereas solo tasks were used in previous experiments^15–17^. Another distinction from previous studies is that the required synchronization rate was set equal to the mean of each pair’s solo production rates, to equate difficulty across individuals.

Nonmusicians showed less flexibility than musicians in synchronization recovery from the delayed auditory feedback interventions. Nonmusician participants who had the delayed feedback intervention first showed greater variability than all other participants, including nonmusicians who had the normal feedback intervention first. Nonmusician participants who had the delayed feedback intervention first were more variable when synchronizing in the turn-tasking than all other participants, including nonmusicians who had the normal feedback intervention first. This finding is consistent with two previous discoveries: that musicians are less affected by altered feedback than nonmusicians^15–17^ and that musicians adapt more flexibly to temporal perturbations than nonmusicians^10,46^. Thus, the effect of the delayed feedback intervention lasted longer for nonmusicians than for musicians. Because the synchronization rate was set to the mean of each pair’s solo production rates, obtained differences between musically trained and untrained pairs could not be attributed solely to rate differences.

Synchronizing one’s movement to rhythmic stimuli requires attentional resources, especially when responding to perturbations^47^. The delayed feedback intervention conditions may have disrupted participants’ attentional processes, resulting in disturbed rhythmic coordination with their partner, and more so in nonmusicians who allocate more cognitive resources to maintain synchronization^47^. Future studies are necessary to investigate the effect of attentional resources on participants’ interpersonal synchronization, for example using dual-task paradigms.

Partners’ social interaction ratings were lower in perceived connectedness, relationship, and synchrony following the delayed auditory feedback interventions than the normal auditory feedback intervention. Participants reported smaller relationship scores after the delayed feedback intervention, but this effect was mostly driven by nonmusician participants who received the delayed feedback condition first. These results are in line with previous studies that showed that action synchronicity increases social connections and interpersonal liking^23–25,48^. A possible caveat is that social interaction influences from temporal perturbations may be harder to manipulate in musicians who are trained to overcome synchronization differences in joint performance.

Finally, a delay-coupled model was fit to the participants’ asynchronies in the turn-taking tasks. Results showed that, overall, coupling strength was larger in musicians. Nonmusicians who did the delayed feedback condition first had the lowest coupling strength, corroborating the behavioral synchronization results and the relationship ratings. Coupling strength correlated with participants’ asynchronies, confirming that the estimated parameters captured the observed behaviour. In addition, there was a large range of SPRs across participants, and a large range of differences between the SPRs of randomly paired participants. Positive correlations between participants SPRs (expressed as the difference between their tapping rate and the metronome rate) and coupling strength indicated that in general, participants with slower spontaneous tapping rates coupled more. This aligns with previous findings that individuals with faster intrinsic frequencies tend to be less flexible (thus showing less coupling) than slower individuals in adjusting to a different production rate^10^.

To conclude, short-term synchrony interventions that altered auditory feedback causally affected musicians’ and nonmusicians’ perceived social interaction and subsequent synchronization abilities. These findings confirm that social connection with a partner depends on the degree of synchronization experienced. A dynamical system’s oscillator (delay-coupled) model accounted for the asynchronies in the turn-taking condition. The coupling term of the model captured the differences in social interaction and synchronization asynchronies resulting from the intervention. This study shows a clear carryover effect of altered auditory feedback intervention on subsequent turn-taking synchronization, and that its reversal can be established in subsequent interventions. Overall, these findings suggest that interventions that disrupt temporal coordination with a partner affect not only social connectedness between partners but also their ability to stay synchronous during subsequent turn-taking tasks.

## Data Availability

The data are available at https://osf.io/bnzmr/.
